# Branched xenopericardial roll graft replacement of an infected aortic arch graft

**DOI:** 10.1111/jocs.13986

**Published:** 2019-01-09

**Authors:** Hiroshi Kubota, Hidehito Endo, Hikaru Ishii, Hiroshi Tsuchiya, Norihiko Ohura, Yu Takahashi

**Affiliations:** ^1^ Department of Cardiovascular Surgery Kyorin University Tokyo Japan; ^2^ Department of Plastic Reconstructive and Aesthetic Surgery Kyorin University Tokyo Japan; ^3^ Department of Cardiovascular Surgery National Disaster Medical Center Tachikawa Japan

**Keywords:** aorta and great vessels, aortic arch, aortic infection, graft infection, xenopericardium, xenopericardial roll graft

## Abstract

Which graft material is the optimal graft material for the treatment of aortic graft infections is still a matter of controversy. We used a branched xenopericardial roll graft to replace an infected aortic arch graft as a “rescue” operation. The patient is alive and well 37 months postoperatively without recurrence of the infection and any surgical complication. This procedure may have the possibility to serve as an option for the treatment of aortic arch graft infection.

## 1 INTRODUCTION

The optimal graft material for the surgical treatment of aortic graft infections is still a matter of controversy. Orthotopic aortic reconstruction with an intraoperatively prepared xenopericardial branched roll graft was successfully performed as a rescue operation to treat a patient with a graft infection.

### 1.1 Patient profile

**Figure 1:**
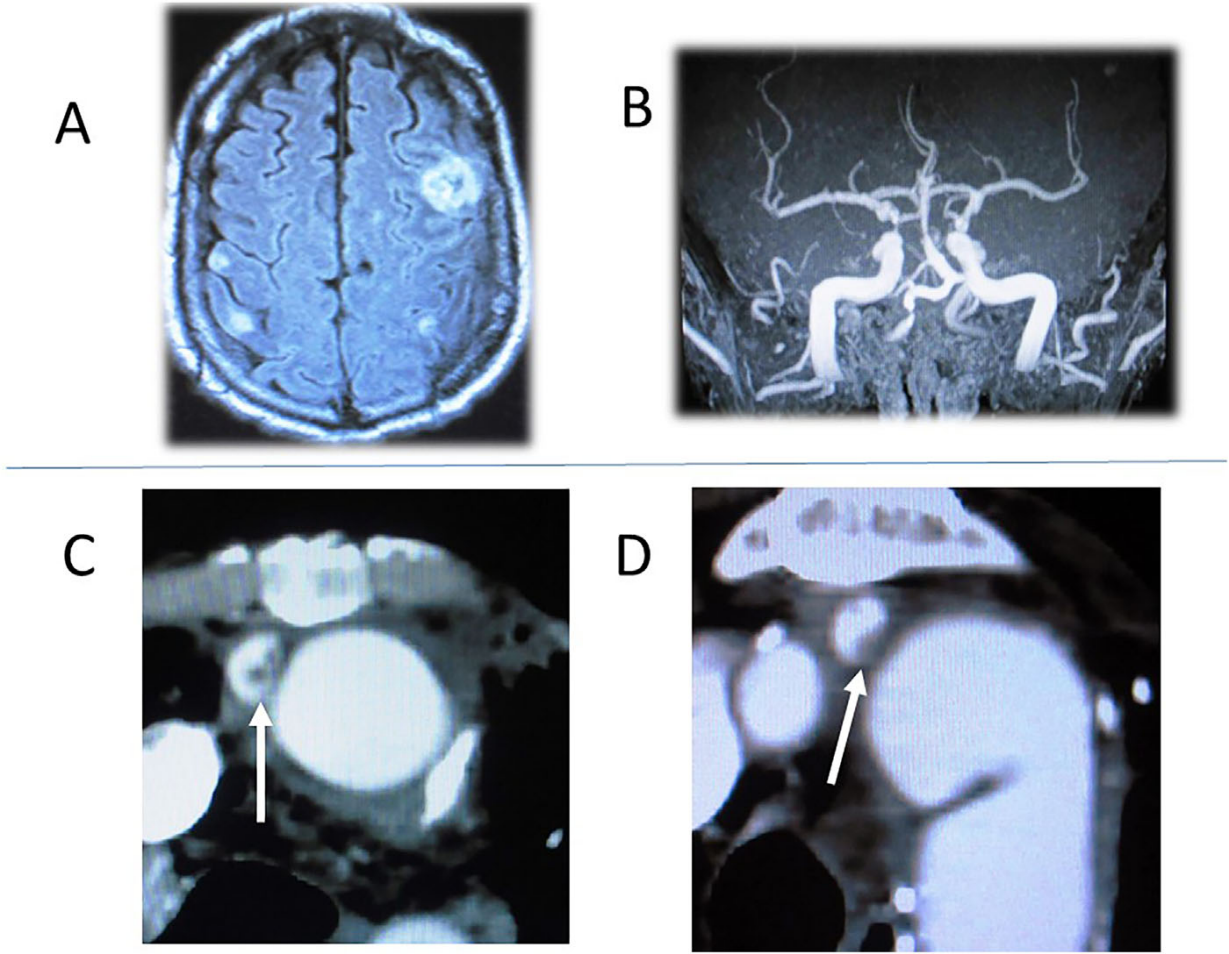
Preoperative magnetic resonance image and enhanced computed tomography. A, Multiple fresh mycotic brain infarctions were detected. B, Severe stenosis of the right internal carotid artery was detected. C, Vegetation was detected in the brachiocephalic graft branch. D, Vegetation was detected in the left common carotid branch

A 66‐year‐old male was urgently transferred to our hospital because of fever, general malaise, and refractory skin to graft fistula after a total aortic arch replacement in 2015. He had a complicated past history of treatment for major cardiac and cerebrovascular disease. He had undergone a percutaneous coronary intervention (PCI) for acute myocardial infarction at 55 years of age, and coronary artery bypass graft surgery x4 using left internal thoracic artery, radial artery, and saphenous vein grafts at 56 years of age. In 2012, at 63 years of age, a triplex 26 mm (Terumo, Tokyo, Japan) had been used to perform a total aortic arch replacement procedure to treat a true aortic arch aneurysm at a nearby hospital. A reversed elephant trunk was inserted to facilitate the distal anastomosis using stepwise distal anastomosis technique. Preoperative coronary angiography showed the following stenosis: proximal left anterior descending artery, 75%; 1st diagonal branch, 75%; 1st left posterolateral branch, 75%; and 2^nd^ left posterolateral branch, 90%. All coronary artery bypass grafts were occluded. The origin of the left subclavian artery was ligated, and a 3rd graft branch was extended by a graft to the axillary artery via the left thoracic cavity. One month postoperatively, open‐chest drainage had been performed to treat left thoracic empyema. Subsequently, an incisional abscess was noted around the site of the left axillary anastomosis, and a graft‐branch‐to‐skin fistula was diagnosed. In 2013, at 64 years of age, the abscess was opened and the graft branch to the axillary artery was partially removed through a subaxillary skin incision. On admission to our hospital, the patient's serum C‐reactive protein level was elevated to 22.5 mg/dL, and a blood culture was positive for methicillin‐sensitive *Staphylococcus aureus*. The patient gradually became drowsy, and brain magnetic resonance imaging revealed multiple fresh cerebral infarctions and severe right internal carotid artery stenosis (Figure 1A and B). Enhanced computed tomography showed vegetations inside the origin of the graft branch to the brachiocephalic artery and the left common carotid artery (Figure 1C and D). Graft infection, sepsis, and multiple mycotic brain infarctions were diagnosed, and emergency surgery was planned. Written informed consent with the approval of our institutional review board was obtained from the patient's family before performing the procedure.

### 1.2 Surgical procedure

**Figure 2:**
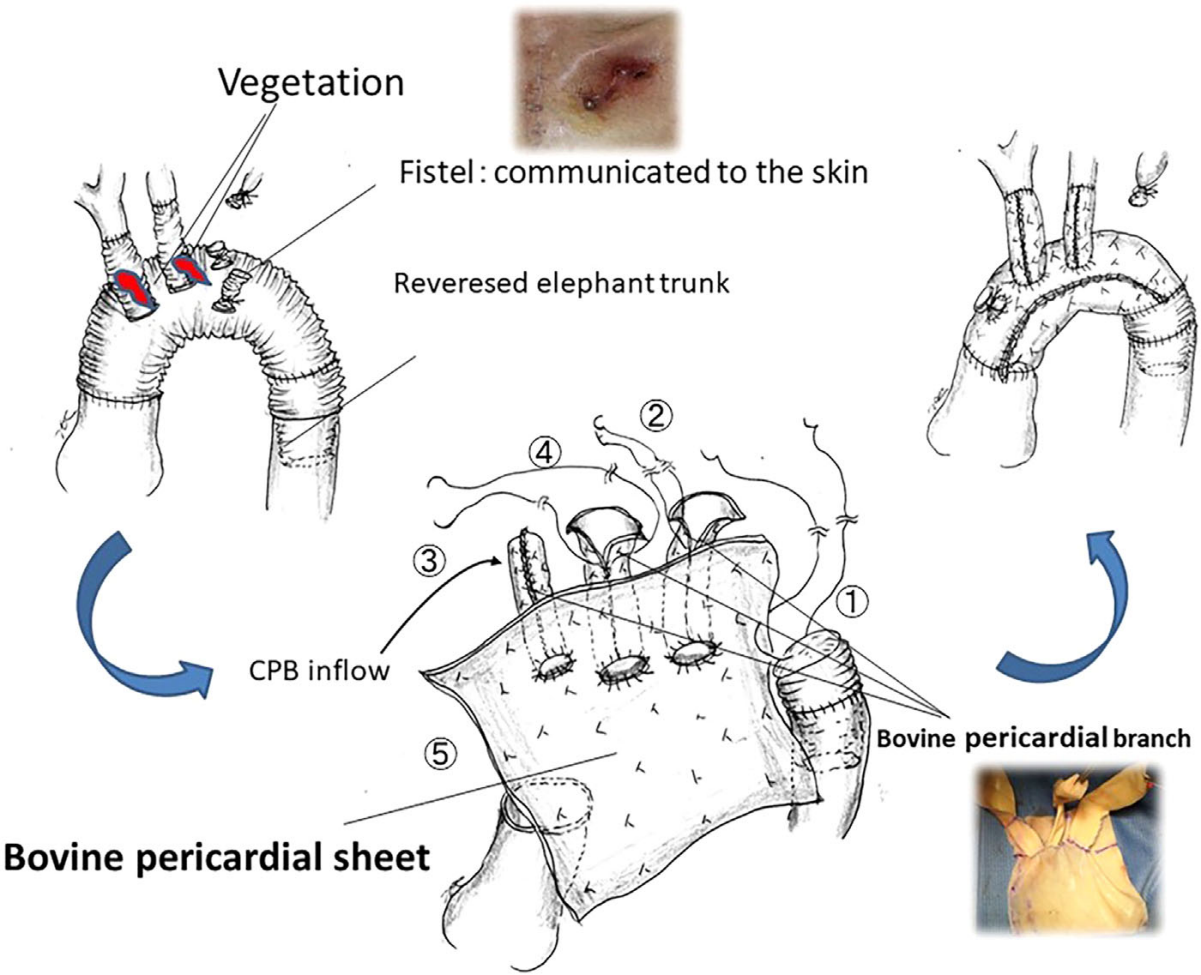
Schema and steps in the surgical procedure. An infected graft was removed and in situ branched pericardial roll graft replacement was performed to create a neo‐aorta. 1) Distal anastomosis. A branched xenopericardial sheet is formed into a cylinder to enable cross‐clamping after step 4. 2) Anastomosis of the left common carotid artery. 3) Antegrade perfusion. 4) Anastomosis of the innominate artery. 5) Proximal anastomosis

A cardiopulmonary bypass through a re‐resternotomy was established by means of two venous cannulas and an inflow cannula into the graft. The left subclavian abscess cavity communicated with the graft. Because of the dense adhesions, the left‐side of the heart was not dissected, and the coronary artery bypass graft was abandoned. Under deep hypothermic circulatory arrest with intermittent pressure‐augmented retrograde cerebral perfusion^1^, ^2^), the graft and branches were removed. Myocardial protection was provided by retrograde cardioplegia with continuous cold blood perfusion. Vegetations were found inside the root of the graft branches. Because the reversed elephant trunk was tightly adherent to the surrounding tissue, it was resected as deeply as possible, but part of it was left intact. A 3‐branched bovine rectangular 10 × 10‐cm^2^ pericardial sheet (XGA‐400; Edwards Lifesciences, Irvine, CA, USA) was simultaneously prepared intraoperatively from bovine pericardial sheets on a separate, sterile operating table. It takes 30‐40 min. to prepare a branched xenopericardial sheet. The method used to prepare the branched xenopericardial roll graft has been described in detail previously^3^). The felts and threads that had been used to close the orifice of the left subclavian artery were removed, and the stump was re‐closed by suturing pericardial patches. One side of the pericardial sheet was anastomosed to the remaining graft with a 4‐0 polypropylene single continuous suture and shaped into a cylinder. The third branch was anastomosed to the left common carotid artery. The first branch was used as the inflow route for the cardiopulmonary bypass. The main graft was clamped, and antegrade perfusion was restored. The second branch was anastomosed to the brachiocephalic artery. After completing the proximal anastomosis, the aorta was de‐clamped. Although weaning from the cardiopulmonary bypass was achieved without difficulty, sudden ventricular fibrillation due to myocardial ischemia occurred during hemostasis. Direct cardiac massage for 9 min and an intra‐aortic balloon pump insertion were required to restore stable vital signs. The chest was closed without omental flap plombage. A sternocleidomastoid muscle pedicled flap was used to fill the subclavian cavity (Figure 2). Circulatory arrest time was 80 min; aortic cross‐clamp time was 144 min, and total perfusion time was 239 min. Minimum tympanic membrane temperature was 19.6°C (Video). On postoperative day 4, percutaneous balloon dilatation was performed to treat the severe stenosis of the elephant trunk, which had became tortuous as a result of having tried to extract it intraoperatively. On postoperative day 22, the C‐reactive protein level was within the normal range, and the patient's consciousness had recovered well without neurological deficit. On postoperative day 45, the patient was transferred to another hospital. After 5 months of rehabilitation, he was discharged unassisted. One year postoperatively oral antibiotic administration was discontinued. We considered immediate PCI after the operation; however, since the patient required renal replacement therapy for 9 days, and there were no angina symptoms or electrocardiogram changes during rehabilitation, we hesitated to perform an immediate PCI, and selected elective coronary angiography and PCI after mild symptoms appeared. In retrospect, ventricular fibrillation that developed during the operation was induced not only by the coronary artery stenosis but by hemodynamic instability due to the long perfusion time and operative invasiveness. Two years after the operation, percutaneous coronary artery intervention was performed to treat unstable angina pectoris caused by stenosis of the left anterior descending artery and the left circumflex artery.

**Figure 3:**
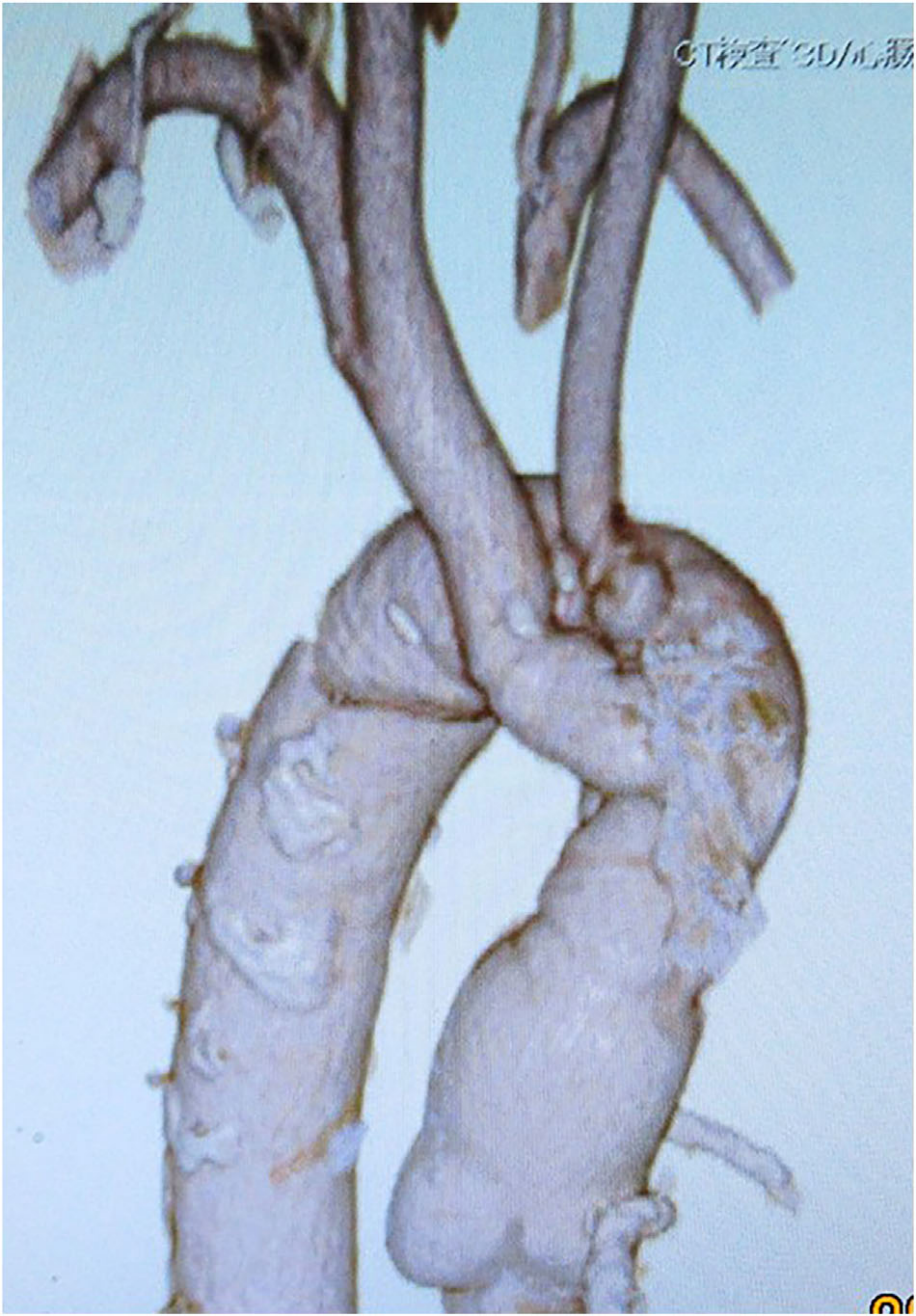
Postoperative 3‐dimensional computed tomography image acquired 36 months postoperatively. There are no stenoses, no calcifications, no thrombus formation, and no pseudoaneurysms of the neo‐aorta or its branches

As of 37 months postoperatively, the patient has been free of local recurrences of the graft infection, of pseudoaneurysms on the suture lines, and of other graft‐related complications, including of the branches (Figure 3).

## 2 DISCUSSION

Complete removal of the infected graft and in situ replacement with rifampicin‐soaked Dacron grafts is the standard procedure to cure graft infection. A rerouting technique, not an in situ technique, is another option^4^). Although cryopreserved arterial homografts are one of the types of materials used to treat infected aortas, the supply in Japan is inadequate, and it is difficult to obtain one in time for an urgent operation. We previously reported eight cases of xenopericardial roll graft replacement to treat aortic/graft infection^5^), and here we have reported the 9th case.

Czerny et al. found that treatment of graft infections after surgical or endovascular treatment of thoracic aortic disease by complete removal of the infected prosthetic material and orthotopic vascular reconstruction by using xenopericardial tube grafts as neoaortic segments provided excellent results with regard to durability and freedom from reinfection and reoperation^6^). Pericardial sheets are soft and easy to handle. They are shaped into cylinders intraoperatively by rolling them up, and have provided us with a good operative field.

In contrast to simple tube grafts, we developed a branched xenopericardial tube graft. In our first case, even though it was an elective operation, we decided to prepare a branched xenopericardial sheet intraoperatively, because the native aortic infection site appeared to be wider and deeper than expected, rather than being located in just the ascending aorta.

Major concerns in regard to separately reconstructed neck vessels are graft stenosis, calcification, and possible thrombus formation. We routinely administer an anticoagulant or antiplatelet drug to prevent branch‐related complications. We have performed six aortic arch reconstructions by using branched xenopericardial sheets thus far. All of the cases except the 1st were treated by “rescue” operations that involved removing the infected graft. The patient in the 1st case died of lung cancer 45 months postoperatively, and the other five patients, including the 2nd patient in the case presented here, are alive and well as of 2, 16, 19, 24, and 37 months postoperatively and have been free of local recurrence of the infection, of graft calcification, of pseudoaneurysm on suture line, and of other graft‐related complications, including of the branches. Using a bovine bifurcation prosthesis (Biological Bifurcation Conduit, Synovis, Life Technologies, St. Paul, MN) together with a xenopericardial sheet may be another option to achieve partial reconstruction of the aortic arch^7^). Hostalrich et al, systematically reviewed the literature on xenopericardial graft reconstruction as a means of treating aortic/graft infections and reported finding that the technical success rate was 100% in a total of four studies describing 71 cases. Mean 30‐day mortality was 25%, and only one death (1.4%) was linked to an operator‐prepared pericardial tube graft^8^).

In conclusion, accumulation of clinical cases and confirmation of the long‐term durability of branched xenopericardial grafts may demonstrate their advantage as one of the options for the treatment for native aortic and aortic arch graft infection.

## Supporting information

Additional supporting information may be found online in the Supporting Information section at the end of the article.

Supplementary Video

## References

[1] Kubota H , Tonari K , Endo H , Tsuchiya H , Yoshino H , Sudo K . Total aortic arch replacement under intermittent pressure‐augmented retrograde cerebral perfusion. J Cardiothorac Surg. 2010; 5:97. 21044311 10.1186/1749-8090-5-97 PMC2987928

[2] Endo H , Kubota H , Tsuchiya H , et al. Clinical efficacy of intermittent pressure augmented‐retrograde cerebral perfusion. J Thorac Cardiovasc Surg. 2013; 145:768–773. 22498084 10.1016/j.jtcvs.2012.03.015

[3] Kubota H , Endo H , Noma M , et al. Equine pericardial roll graft replacement of infected pseudoaneurysm of the aortic arch. J Cardiothorac Surg. 2012; 7:45. 22583570 10.1186/1749-8090-7-45 PMC3418200

[4] Bianco V , Kilic A , Gleason TG , et al. Management of thoracic aortic graft infections. J Card Surg. 2018; 33:658–665. 30178475 10.1111/jocs.13792

[5] Kubota H , Endo H , Noma M , et al. Xenopericardial roll graft replacement for infectious pseudoaneurysms and graft infections of the aorta. J Cardiothorac Surg. 2015; 10:133. 26506850 10.1186/s13019-015-0343-5 PMC4624649

[6] Czerny M , Allen R , Opfermann P , et al. Self‐made pericardial tube graft: a new surgical concept for treatment of graft infections after thoracic and abdominal aortic procedures. Ann Thorac Surg. 2011; 92:1657–1662. 21945229 10.1016/j.athoracsur.2011.06.073

[7] Hoff AHT , Akca F , Cuypers PWM , et al. Mycotic innominate artery aneurysm repair using a bovine pericardial bifurcation prosthesis. J Card Surg. 2018; 33:146–148. 29526047 10.1111/jocs.13553

[8] Hostalrich A , Ozdemir BA , Sfeir J , et al. Systematic review of native and graft‐related aortic infection outcome managed with orthotopic xenopericardial grafts. J Vasc Surg. 2018; in press: 10.1016/j.jvs.2018.07.072. [Epub ahead of print]. 30528399

